# Evaluating the impact of personalized rehabilitation nursing intervention on postoperative recovery of respiratory function among thoracic surgery intensive care unit patients: A protocol for systematic review and meta-analysis

**DOI:** 10.1097/MD.0000000000028494

**Published:** 2022-01-21

**Authors:** Chun-Yan Li, Wen Liang, Yan-Qin He, Qun Han

**Affiliations:** The First People's Hospital of Jiangxia District, Wuhan City, Hubei Province, China.

**Keywords:** intensive care unit, personalized rehabilitation nursing, respiratory function, thoracic surgery

## Abstract

**Background:**

Exercise tolerance and lung function can be improved by pulmonary rehabilitation. As a result, it may lower thoracic surgery intensive care unit (ICU) patients’ postoperative problems and death. Enhanced recovery after surgery has advanced significantly in the perioperative care of thoracic surgery ICU patients in recent years, and it now plays an essential role in improving ICU patients’ postoperative prognosis. Appropriate tailored rehabilitation nursing intervention is required to promote the postoperative recovery of respiratory function in thoracic surgery ICU patients. This study aims to look at the influence of tailored rehabilitation nurse intervention on postoperative respiratory function recovery in thoracic surgery ICU patients.

**Methods:**

To find relevant papers, a comprehensive search of electronic databases will be conducted, including three English databases (PubMed, EMBASE, and the Cochrane Library) and two Chinese databases (Chinese National Knowledge Infrastructure and WanFang). Only research that has been published in either English or Chinese will be considered. The retrieval period will run from November 2021 to November 2021. We will look at randomized controlled trials (RCTs) studies that looked at the effect of a customized rehabilitation nursing intervention on the recovery of respiratory function in thoracic surgery ICU patients after surgery. Two writers will review the literature, retrieve study data, and assess the included studies’ quality. Any disagreements will be settled via consensus. RevMan 5.3 will be used to do the meta-analysis.

**Results:**

This research will offer high-quality data on the influence of customized rehabilitation nurse intervention on postoperative respiratory function recovery in thoracic surgery ICU patients.

**Conclusion:**

This study will look at whether a targeted rehabilitation nurse intervention might help thoracic surgery ICU patients recover their respiratory function more quickly after surgery.

**Ethics and dissemination:**

There will be no need for ethical approval.

**Registration number:**

December 12, 2021.osf.io/9rdu2/ (https://osf.io/9rdu2/).

## 1 Introduction

According to a 1997 American Thoracic Society explanation on ICU distribution choices, the essential objective of the emergency unit is to “safeguard significant human existence by ensuring and supporting patients in a mindful way when they are compromised by an intense basic disease or injury or because of clinical or surgical treatment.”^[1]^ The ICU is utilized to screen and really focus on patients who might have physiological unsteadiness and require specialized or potentially life support, as indicated by the 1999 update.^[2]^ Just a minuscule level of patients admitted to the ICU after thoracic medical procedure foster intense issues that require prompt consideration; the rest are admitted for “monitoring” of likely clinical weakening or essentially for observing.^[3,4]^ Patients who are admitted to the ICU just for observation may have complications such as increased pressure due to the weather, as well as rest and family stress. Furthermore, the cost of medical services has skyrocketed.^[5]^

Patients having critical thoracic surgery must prevent bad effects on the heart, lungs, and other organs, as well as unfavourable responses such as blood circulation problems and respiratory insufficiency, and must be well cared for.^[6]^ It is vital to identify likely to correspond to appropriate nursing action to decrease adverse effects in thoracic surgery ICU patients to promote postoperative recovery of respiratory function. Traditional nursing comprises systematic teaching and discharges counselling of disease-related information during hospitalization, and it ends patient health guidance after discharge. Because the demand for medical standards and services is growing as people's living conditions rise, the traditional nursing model has been unable to satisfy the needs of most patients.^[7]^ Rehabilitation tailored to the individual Nursing is a new nursing model that starts with the patient's specific condition and needs, gathers clinical evidence by consulting literature, formulates a scientific and reasonable nursing plan, and adjusts it over time based on the effectiveness of nursing measures to achieve the best nursing effect.

The review that will be written following this procedure will include scientific information on the impact of personalized rehabilitation nursing intervention on postoperative recovery of respiratory function in thoracic surgery ICU patients.

## 2 Objectives

This meta-analysis and systematic review procedure examine how customized rehabilitation nursing care affects postoperative respiratory function recovery in thoracic surgery ICU patients.

## 3 Methods

The Open Science Framework (https://osf.io/) reviewed and approved this protocol. The Preferred Reporting Items for Systematic Reviews and Meta-analyses statement will be followed in this research.^[8]^

### 3.1 Eligibility criteria

#### 3.1.1 Types of studies

The English and Chinese languages will interpret the randomized controlled trials (RCTs). Animal studies and studies with missing data will be disqualified.

#### 3.1.2 Types of participants

Patients undergoing thoracic surgery ICU care will be included without gender or nationality.

#### 3.1.3 Types of interventions

The following criteria will be satisfied by including relevant RCTs:

(1) in the experiment group, employing tailored rehabilitative nursing intervention alone or in conjunction with another nursing intervention;
(2) in the control group, nursing intervention other than individualized rehabilitative nursing or no nursing intervention.

#### 3.1.4 Types of outcomes measures

FEV1, FVC, FEV1/FVC, heart rate, respiratory frequency, nurse satisfaction, the incidence of adverse reactions and major events, and the length of hospital stay are all outcomes.

### 3.2 Search methods for the identification of studies

A comprehensive search of electronic databases will be conducted to find relevant papers, including three English databases (PubMed, EMBASE, and the Cochrane Library) and two Chinese databases (Chinese National Knowledge Infrastructure and WanFang). Only research that has been published in either English or Chinese will be considered. The retrieval period will run from November 2021 to November 2021. The retrieval mode will be a combination of free words and medical subject headings, such as “intensive care unit, personalized rehabilitation nursing, randomized controlled trials, and so on.”

### 3.3 Data collection and analysis

#### 3.3.1 Study selection

**Figure 1:**
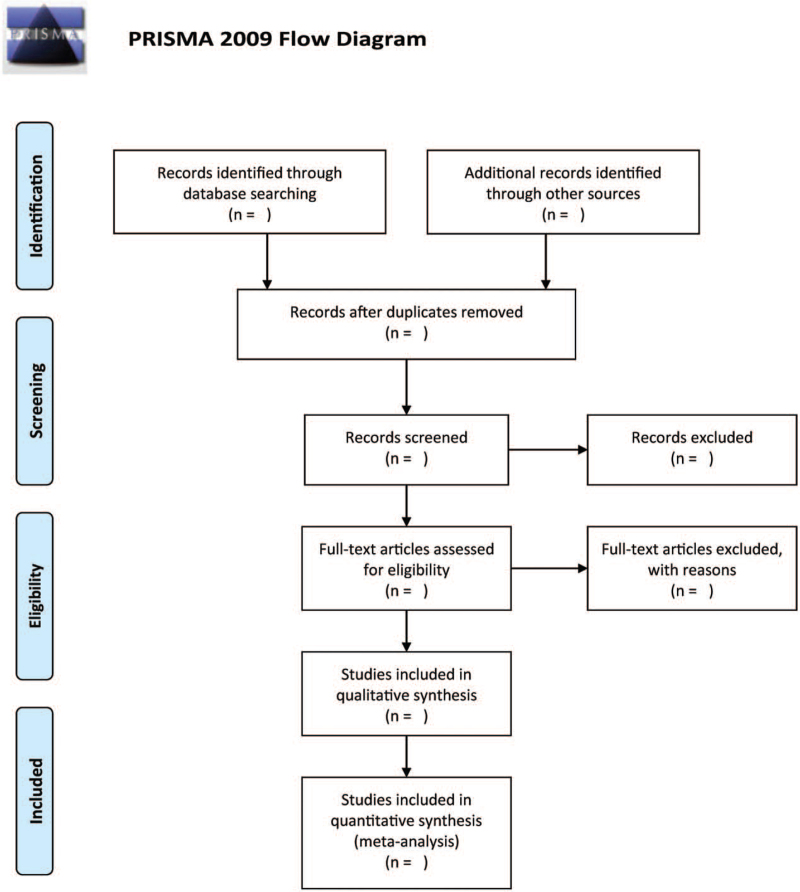
The research flowchart.

We will search the databases for all relevant literature using the right topic phrases and keywords, then delete duplicate papers. Then, two separate writers will go over the title and abstract of the literature to exclude those that are not relevant to this study. Then, using the previously devised principles of eligibility criteria, read the whole text of the remaining literature to decide the eligible literature. Any differences in study selection will be handled by discussion or consultation with the third author. Figure 1 depicts a flow diagram of the selection process.

#### 3.3.2 Data extraction

Two authors from the database for data extraction and management, including title, year of publication, first author's name, general information, study design, research cycle, sample size, experimental and control group intervention techniques, results, and adverse events. Any differences in study selection will be handled by discussion or consultation with the third author.

#### 3.3.3 Assessment of the risk of bias

Two authors will independently assess the risk of bias, including random sequence generation, allocation concealment, blinding, inadequate outcome data, selective outcome reporting, and other biases, using the Cochrane assessment of the risk of bias (V.5.1.0).^[9]^ High, low, and uncertain will be the judgements of assessed domains. Any differences in study selection will be handled by discussion or consultation with the third author.

#### 3.3.4 Dealing with missing data

We will contact the respective author if comprehensive literature or relevant data is not accessible. The study will be removed from consideration if the missing data cannot be provided.

#### 3.3.5 Measures of treatment effect

For dichotomous outcomes (CI), risk ratios with 95 percent confidence intervals will be reported for dichotomous outcomes (CI). Mean differences or standardized mean differences will be calculated where continuous outcomes exist.

#### 3.3.6 Assessment of heterogeneity

According to the Cochrane Handbook for Systematic Reviews of Interventions, we will utilize the *I*^2^ statistic to analyze heterogeneity and quantify inconsistency in all included studies. Significant heterogeneity is indicated when the *I*^2^ score is larger than 50%.

#### 3.3.7 Sensitivity analysis

To test the reliability and robustness of the aggregation findings, a sensitivity analysis of each parameter was performed using the one-by-one elimination technique. The findings of the sensitivity assessments will be reported in a summary table.

#### 3.3.8 Publication bias

If there are more than ten articles, a funnel plot will be utilized to indicate publishing bias due to its symmetry.

## 4 Discussion

This review strategy was utilized to conduct a systematic review and meta-analysis to determine the influence of individualized rehabilitation nursing intervention on postoperative respiratory function recovery in thoracic surgery ICU patients. It explains the trial's kind, participants, interventions, and outcomes, as well as search techniques, data sources, extraction methods, and data analysis. Only published research will be included in our search approach.

There are several limits to our review that we should be aware of. First, owing to the constraints of English and Chinese studies, there may be linguistic prejudice. Second, because the research populations’ baseline for each trial and the study design vary, some variations may be among trials. When there is the heterogeneity, subgroup and meta-regression analysis will investigate the likely causes. Our findings will provide scientific evidence for high-quality, scientific, and effective nursing intervention on postoperative recovery of respiratory function in thoracic surgery ICU patients, which is critical for reducing complications, improving nursing satisfaction, and improving patient quality of life.

## Author contributions

**Conceptualization:** Chun-Yan Li, Yanqin He, Qun Han.

**Data curation:** Chun-Yan Li, Wen Liang, Qun Han.

**Formal analysis:** Chun-Yan Li, Yanqin He, Qun Han.

**Funding acquisition:** Chun-Yan Li, Wen Liang.

**Investigation:** Yanqin He, Qun Han.

**Methodology:** Chun-Yan Li, Wen Liang.

**Project administration:** Chun-Yan Li, Wen Liang, Yanqin He.

**Resources:** Chun-Yan Li, Wen Liang, Yanqin He, Qun Han.

**Software:** Chun-Yan Li, Wen Liang, Yanqin He, Qun Han.

**Validation:** Chun-Yan Li, Wen Liang.

**Visualization:** Chun-Yan Li, Wen Liang, Yanqin He.

**Writing – original draft:** Chun-Yan Li, Yanqin He, Qun Han.

**Writing – review & editing:** Qun Han.
